# Verification of experimental results with simulation on production of few-layer graphene by liquid-phase exfoliation utilizing sonication

**DOI:** 10.1038/s41598-022-10971-w

**Published:** 2022-06-14

**Authors:** Sayed Waliulhaq Mushfiq, Reza Afzalzadeh

**Affiliations:** Faculty of Physics, K. N. Toosi University of Technology, Tehran, 15418-49611 Iran

**Keywords:** Materials science, Nanoscience and technology, Physics

## Abstract

Graphene, an extraordinary tow-dimensional carbon nanostructure, has attracted global attention due to its electronic, mechanical, and chemical properties; therefore, there is a need to find out an economical mass production method to produce graphene. In the present research, the aim is to find out optimal conditions for exfoliation of few-layers graphene (FLG) in a water–ethanol green solution. We varied different parameters of the ultrasonic probe like power quantity and time duration of sonication to investigate the effects on the number of graphene layers and density of graphene in the solution. Also, an attempt has been made to predict the acoustic pressure distribution by solving the wave equation in various output powers of the ultrasonic probe (sonotrode) using numerical simulations. The simulations and experimentations verify each other. Concluding that modifying the output power at the same condition will significantly alter the acoustic pressure inside the sonoreactor. The difference in acoustic pressure at 90% output power of our experimentations is much higher than in other conditions. Experimentation results utilizing UV–visible spectra, SEM (Scanning electron microscope), TEM (Transmission electron microscope) images and Raman spectrum indicate that the minimum thickness and maximum exfoliation for these samples are acquired for sonication at 90% of the maximum effective output power of the sonicator being 264 W for 55 min.

## Introduction

Recently, graphene as a single atomic layer of carbon has attracted significant attention for research due to its unique nanostructure, mechanical, electric and novel thermal properties^1–5^. The firstly pioneering experiments have been carried out on micro mechanically cleaved monolayers, but micro mechanical method has the disadvantages of low production yield and poor throughput. In order to utilize graphene for future industrial applications requiring large-scale and high-output processing methods are required^6^. At present, the reduction of graphene oxide is the preferred scalable method for the preparation of graphene. In this method, the oxidization of graphite by following Hummers modified method, followed by exfoliation of graphite oxide in water, to give aqueous dispersions of graphene oxide (GO) and further the oxidation can be removed by thermal or chemical reduction^7,8^. However, the reduction of graphene oxide still retains a high defect density, which degrades their properties.

Most researchers have focused on the direct solution-phase exfoliation of natural graphite flaks to overcome the limitation of graphene oxide. A few layers of graphene were synthesized by evaporating polystyrene at atmospheric pressure by the chemical vapor deposition (APCVD) method^9,10^. Graphene sheets can be extracted through sonication^11^ or shear exfoliation^12^ in an aqueous surfactant solution and certain organic solvents. Several groups have shown that graphene can be prepared in *N*-methyl-2-pyrrolidone^13^ (NMP), and dimethylformamide^14^ (DMF), to produce graphene nano-sheets. These solvents have surface tension close to 40 mJ m^−2^ and are suitable for direct exfoliation of graphite into FLG^15^. However, these solvents have high boiling points that limit their viability for real use. Thus, dispersion of graphene in low boiling point solvents is useful in many applications, such as nanocomposites^16^ nanoplasmonics^17–19^, smart coating^20^, and high-frequency devices^21–23^. Many reports present the effect of sonication parameters on produced MoS_2_ nano-flakes and graphene in DI water-ethanol^24–26^. A simple, low-cost, and energy-effective sonication to exfoliate natural graphite flakes in ethanol in a short time sonication is reported^27^. Studies are aimed at determining the rate of exfoliation of various forms of graphite and its oxide using the top-down method and using UV–Vis spectroscopy for a deeper understanding of the electronic transition are presented^28,29^. In another article, one can find out UV–Vis. method to measure the percentage of few-layer sheets in GO dispersions quickly and cheaply^30^.

In this research study, an attempt has been made to predict the acoustic pressure distribution by solving the wave equation in various output power of the ultrasonic probe. Simulations have been carried out by using a numerical model. Then Graphene is prepared via the LPE (liquid phase exfoliation) method utilizing the ultrasonic probe. Then we investigated the various effects of the output power, sonication time duration for the same concentration to find the optimal condition to achieve more graphene with thinner layer from graphite powder. Then simulations are compared with experimental results.

## Experimentation

In this section material method and the samples preparation are presented.

### Material and methods

All the chemicals used in this project were research-grade and deionized water (DI) throughout the experiments. The used liquid medium is a green solution prepared by a mixture of 65% DI Water and 35% Ethanol. The sonication is carried out using the ultrasonic processor with a titanium probe (Tip) of 22 mm in diameter (FAPAN-1200UPS model made in Iran by FAPAN Co. Ltd.), operation frequency 20 kHz, and nominal power of 1200 W (RMS power of 300 W).

### Sample preparation

Experimentation for all samples is carried by immersing 250 mg of graphite powder added into 125 mL of water–ethanol in a 150 mL glass beaker. We aim to utilize a “green solvent” in order to minimize the environmental impacts. As expected, water is the greenest solvent^31^. But water has a surface tension of about 72 mJ m^−2^. The range of solvent surface tension that can better exfoliate graphene^32^ is 40–50 mJ m^−2^. Optimal surface tension to minimize interfacial tension between graphene and solvent is a mixture of water and a solvent with lower surface tension. Ethanol is a good choice because it has a low boiling point with surface tension close to 22.1 mJ m^−2^. The 65/35 volume ratio for DI water/ethanol is calculated using the Connors-Wright equation with the surface tension of the solution to be 46 m J m^−2^ for making binary aqueous organic solutions^27^. The beaker is kept in an ice-water bath to reduce evaporation of the ethanol due to an increase in the temperature of the solution during the sonication process. Furthermore, the sonicator is adjusted to pulse mode of 50% to prevent overheating. So, the total effective sonication time for preparing the samples is 55 min. The thick flakes are eliminated by centrifugation using a bench top centrifuge: the first step is done for 30 min at 3000 rpm, and then the top 3/4th of the dispersion is retained for the next step, then the rest of the liquid is discarded. Afterward, the second step is carried out again for 15 min at 3600 rpm, and finally, the top 3/4th of the supernatant is collected for the following analyses.

## Simulation of acoustic pressure distribution

In this section, an attempt has been made to predict the acoustic pressure distribution by solving the wave equation in various output power of the ultrasonic probe. Simulations have been carried out by using a numerical model. The Computational Fluid Dynamics is a powerful tool that can be used to optimize the characteristic of the sonoreactor. The present numerical study aims to deliver a detailed explanation and instructions to model the sonoreactor under different operating conditions. In the following section, the computational domain, the physical models, and the boundary conditions will be presented. For such an arrangement, it is usually assumed that the highest local pressure value is reached in the close vicinity of the probe. The intensity $I_{us} (0)$ value is given by the Power $P_{us}$ transferred to the reactor through the probe of the transducer divided by the active surface of this tip $A = \pi r^{2}$,

1 $$I_{us} = \,\frac{{P_{us} }}{A}$$

As the distance from the probe increases, the intensity decrease according to an area into which it is spread in a conical shape. For calculation of the intensity distribution, the distribution of ultrasonic pressure amplitude $p_{0} (r)$ is initially calculated. The acoustic streaming effect in an ultrasonic processing cell is modeled using the non-linear Helmholtz equation^33,34^.

The acoustic pressure can be obtained by solving wave equation. If linear wave propagation is assumed and the shear stress is neglected which is corrected for liquids and gases, the wave equation has the form:

2 $$\nabla \left( {\frac{1}{\rho }\nabla p} \right) - \frac{1}{{\rho c^{2} }}\frac{{\partial^{2} p}}{{\partial t^{2} }} = 0$$

The pressure p is considered time harmonic i.e.,

3 $$p(r,t) = p(r)e^{i\omega t}$$

the space-dependent part of the pressure is the solution of the Helmholtz equation,

4 $$\nabla \left( {\frac{1}{\rho }\nabla p} \right) - \frac{{\omega^{2} }}{{\rho c^{2} }}p = 0$$

where *ω* is the angular frequency.

**Figure 1:**
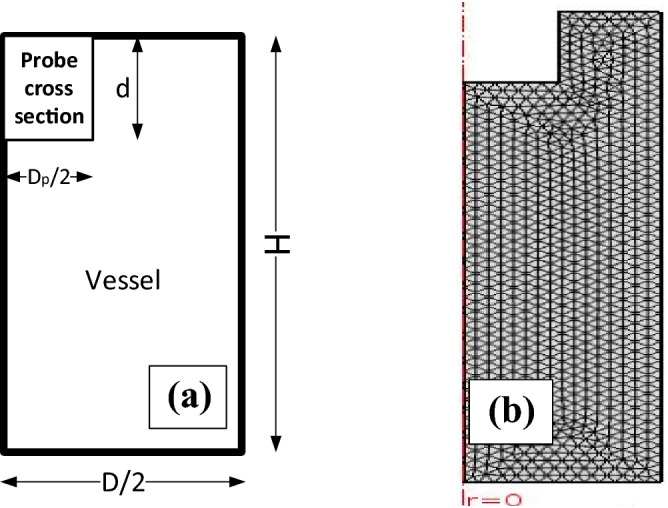
Sonicator and vessel simulation: (**a**) schematic of ultrasonic processing cell with sonotrode (ultrasonic probe of diameter D_p_, immersed depth of probe d, ultrasonic processing cell of diameter D and the height of the liquid inside the cell H); (**b**) scheme of the mesh used for simulation.

An axisymmetric 2D section of the ultrasonic processing cell with an ultrasonic probe is used as the modeling geometry. Figure 1a illustrates the ultrasonic processing cell with the probe and Fig. 1b scheme the mesh used for simulation. Water–ethanol is considered as the fluid medium.

The pressure at the probe tip is calculated from the equation $p = \sqrt {2I\rho c}$ that a relationship between the intensity of the output power to the probe, the density of the medium (ρ), and the corresponding speed of sound (c). The sidewall of the cylinder is modeled as a sound hard boundary. A fine mesh with a node length smaller than the wavelength of the ultrasonic wave is used in the region around the probe tip, while a progressively coarser mesh size is used in the rest of the model^35^.

The acoustic pressure distribution zone in the solution is quantified by estimating the area of the regions within the ultrasonic processing cell. The output power is identified to be influential in determining the size of the resultant acoustic pressure distribution zone. The output power for the validation study is listed in Table 1.

**Table 1:** The real power consumption of 22 mm ultrasonic probe for different percent of output and the calculated radiation intensity.

Max. power (%)	On	20	30	40	50	60	70	80	90
Output power (W)	9.3	34	40	70	171	197	225	245	264
Intensity (W m^−2^)	0.00	8.9 × 10^4^	1.1 × 10^5^	1.8 × 10^5^	4.5 × 10^5^	5.2 × 10^5^	5.9 × 10^5^	6.4× 10^5^	6.9 × 10^5^

It is predicted that this output power would be the most effective among the considered range of parameter for the performance of acoustic pressure distribution for ultrasonic liquid processing. For the acoustic pressure analysis, various output powers were selected and the maximum and minimum acoustic pressure and pressure difference i.e. $\Delta p$ ($\Delta p = P^{ + } - P^{ - }$) are simulated and presented in Table 2.

**Table 2:** Effect of power variation on the maximum and minimum sonicator acoustic pressures and the pressure difference.

Output power (W)	34	40	70	170	197	225	245	264
Max pressure (Pa)	0.48 × 10^6^	0.56 × 10^6^	0 .71 × 10^6^	1.12 × 10^6^	1.2 × 10^6^	1.3 × 10^6^	1.35 × 10^6^	1.4 × 10^6^
Min pressure (Pa)	− 0.43 × 10^6^	− 0.51 × 10^6^	− 0.65 × 10^6^	− 1.0 × 10^6^	−1.1 × 10^6^	− 1.2 × 10^6^	− 1.22 × 10^6^	− 1.3 × 10^6^
Pressure difference (Pa)	0.91 × 10^6^	1.1 × 10^6^	1.4 × 10^6^	2.12 × 10^6^	2.3 × 10^6^	2.5 × 0^6^	2.6 × 10^6^	2.7× 10^6^

### Numerical results

**Figure 2:**
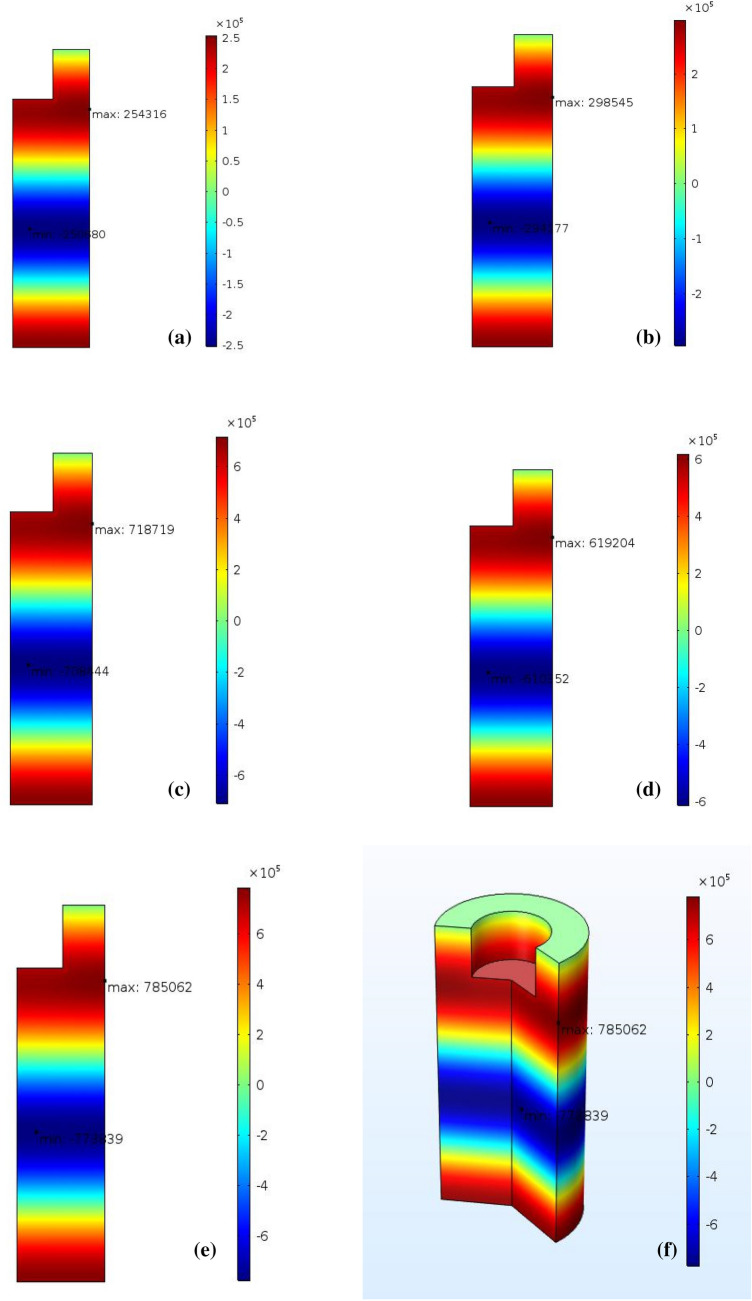
The effect of typical sonicator output power on the acoustic pressure distribution in the vessel: (**a**) 34 W, (**b**) 40 W, (**c**) 170 W, (**d**) 225 W, (**e**) 264 W, 2D, and (**f**) 264 W, 3D analysis.

**Figure 3:**
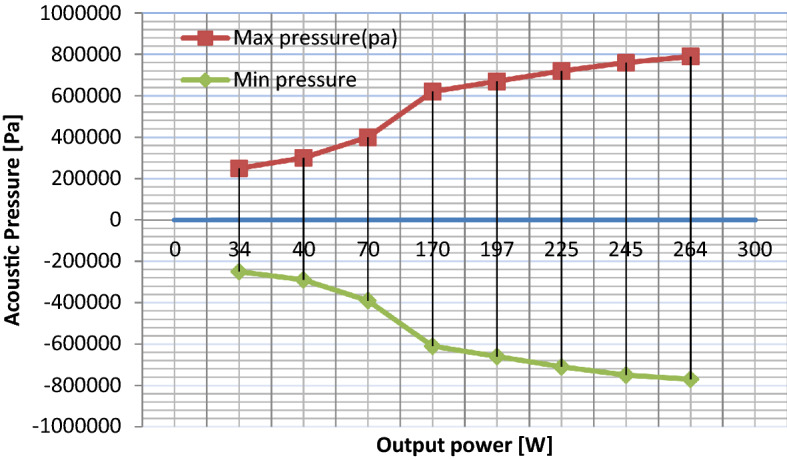
Simulated effect of power variation on the maximum and the minimum probe acoustic pressure in the cylindrical reactor (vessel).

The output power is considered to be one of the critical ultrasonic parameters that significantly affect sonoreactor performance. The impact of the ultrasonic output power on the sonoreactor performance is represented in Fig. 2. At the same conditions, the output power change alters the acoustic pressure range and the wave interactions. We conclude that modifying the output power at the same conditions will significantly alter the maximum and the minimum acoustic pressure inside the sonoreactor. For instance, at 20% (~ 34 W) output power, the maximum pressure is 0.48 × 10^6^ Pa and the minimum pressure is − 0.43 × 10^6^ Pa. While, at 90% of output power (~ 264 W) the maximum pressure is 1.4  × 10^6^ Pa and the minimum pressure is − 1.3 × 10^6^ Pa which is supported by the earlier report^32^. The difference between the maximum and the minimum pressures ($\Delta p$) at 90% of the power is the highest. The minimum and the maximum pressure at various output powers for the 22 mm sonotrode are presented in Fig. 3.

## Results and discussion

In this section, the effects of the varied parameters of the power variation and the time duration of the sonication on the exfoliation process of graphene are presented and discussed.

### Effect of radiation power on the exfoliation

The graphene dispersion is prepared by utilizing the sonicator probe in water–ethanol solution at a various output power of the sonicator with 40%, 50%, 60%, 70%, 80%, and 90% equivalent to 70, 170, 197, 225, 245 and 264 watts (we think that at 70–170 powers, the effect of power on the exfoliation is very small, we gave up testing) in the optimum sonication time of 55 min. From the previous results of the experimentation in this research, the best condition is observed to use a solution with a volume of 125 CC. The effect of changing the output power of sonication in detail by measuring their UV–Vis absorption spectra and SEM are recorded.

**Figure 4:**
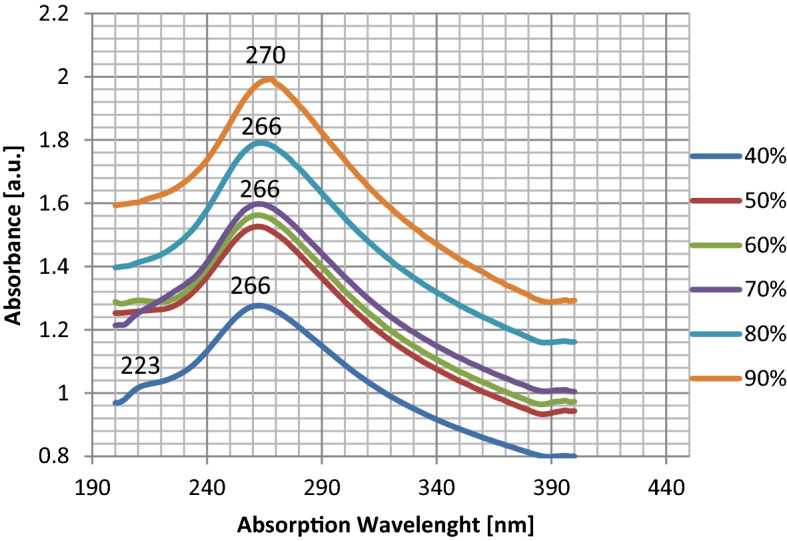
The UV–visible absorption spectrum for the exfoliated graphene in water–ethanol for different sonicating powers.

**Figure 5:**
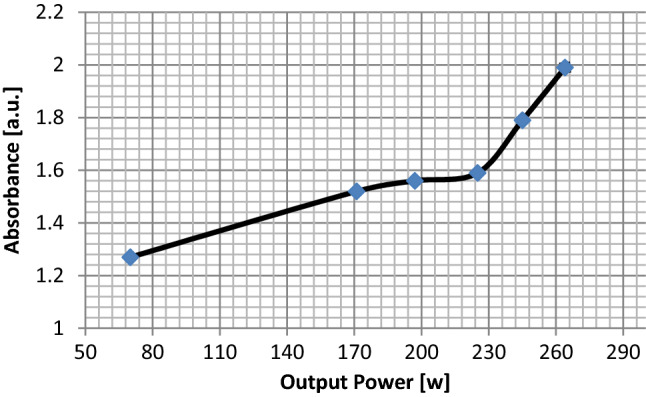
Measurement of the UV–visible absorbance versus power variation for the graphene samples.

The optical characterization of the graphene sheets is performed with a Perkin-Elmer model lambda 25 spectrometer. Using the UV–Vis spectrometer absorption spectrum one can estimate the approximate thickness of the layers or equally the approximate number of layers. The UV–Vis absorption spectra of graphene samples for 40–90% of the maximum power of the continuous radiation mode are shown in Fig. 4. For the sonicated sample with 40% output power, two distinct peaks on 223 nm and 266 nm are indicated which shows the sample contains a mixture of graphene oxide (223 nm peak) and graphene (266 nm peak). Whereas for the cases of 50–90% sonicated power, only one peak is observed with the increase in absorption. In addition, from 50 to 80% sonicated power, the samples show only one peak at 266 nm, while for 90% sonicated power the sample shows an absorption peak at 270 nm. It is expected because of the existence of additional oxygen between layers of graphite oxide, making it easier to exfoliate, thereby having more graphene-like flakes but reduced with oxygen. The UV–Vis absorption spectrum of graphene produced by 90% power of the sonicator is shown in Fig. 4. A prominent peak is found at ≈ 270 nm, corresponding to the π to π^*^ transitions of graphene and graphite^34^. Figure 5 indicates absorption at 270 nm and the relative intensity of the peaks increases with the increased output power from 40% power to 90% power. For the 90% power sonicated sample, a higher amount of few-layer (1–3) graphene compared to the 40% power is observed by an increase in the peak intensity. As can be seen in Fig. 5, there is a continuous increase in absorbance with increasing power.

**Figure 6:**
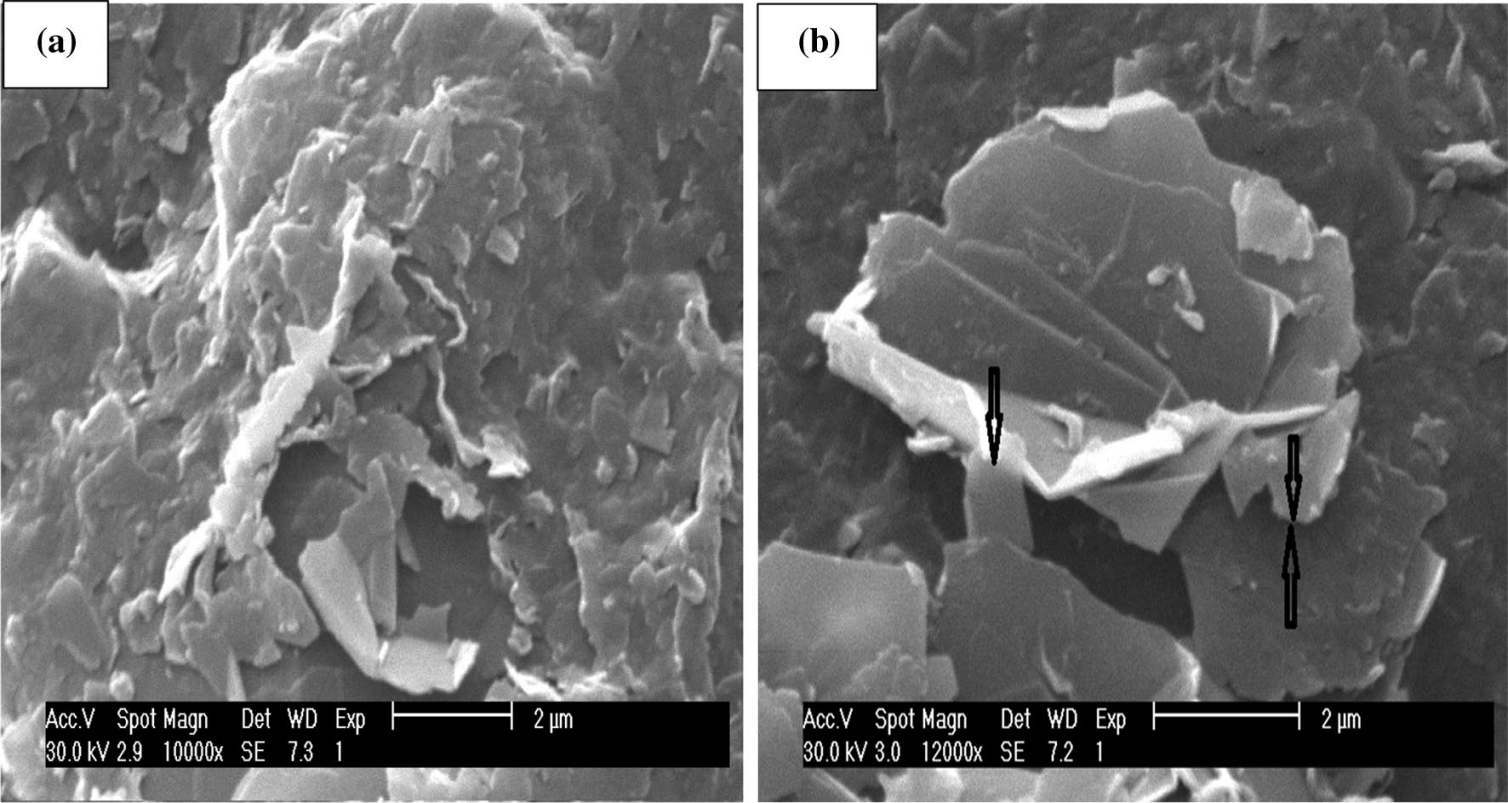
SEM micrographs of the graphene flakes produced at: (**a**) 70 W, (**b**) 264 W sonicator power (scale bars is 2 µm).

This result is also confirmed by SEM (ESEM Philips XL30) images, as shown in Fig. 6. Additionally, Fig. 6 demonstrates that at the power 264 W, flakes' sizes and quality are increased compared to the previous samples. At 70 W, the flakes are smaller and relatively thicker. At 264 W, the dimensions of flakes are about twice the size of the sample exfoliated at 70 W with thinner layers.

#### Comparison of Numerical results and experimentation

From simulation of acoustic pressure distribution, we found that at the same condition^32^, the change of output power alters the acoustic pressure range (Figs. 2, 3). We conclude that modifying output power for the exact geometrical parameters will significantly alter the maximum and the minimum acoustic pressure in the liquid content of the sono-reactor. On the increase in sonicator output power, an increase in positive pressure always equal to negative pressure in the liquid is observed. The difference between the simulated maximum and minimum pressures ($\Delta p$) is increasing by increase in sonicator output power from 0.91 × 10^6^ Pa to 2.7 × 10^6^ Pa (Table 2). An increase in the pressure difference causes the exfoliation of more graphene flakes from graphite powder by overcoming the Van der Waals bond between graphite layers. In addition, at 90% sonicator output, the power shift in absorption to 270 nm (Fig. 5) reveals graphene exfoliation with few layers (1–3 layers), which is supported by an earlier report^34^. On lower power sonication, the graphite multi-layer flakes in the solvent are produced. On the increase in the sonicator power, the exfoliated species convert into few-layer flakes. The simulation verifies experimental results, which by a high increase in ∆p at 90% power achieving few layers, is approved by absorption in 270 nm. The above results from absorption spectra are also approved for a few layers (1–3 layers), multi-layer (4–10 layers), and thick layer (> 10 layers) graphene by previous report^35^.

### Effect of sonication duration on the exfoliation

**Figure 7:**
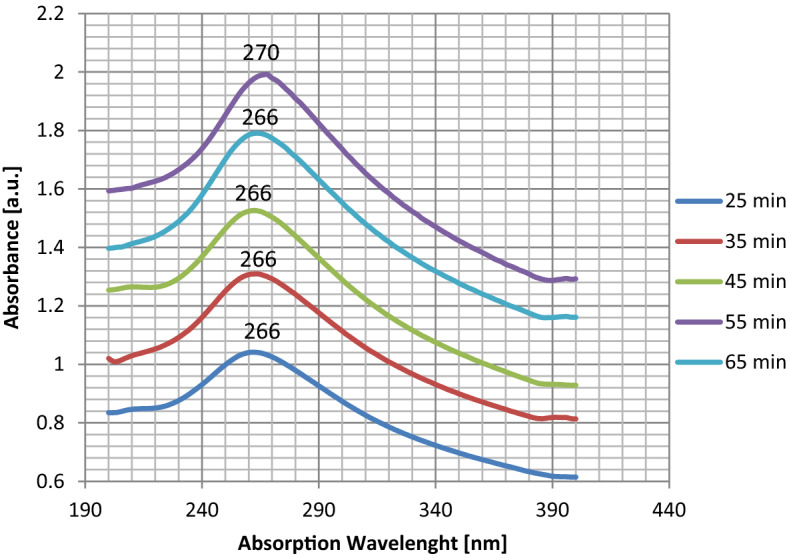
The UV–visible absorption spectrum for the exfoliated graphene for 264 W power versus different sonication time durations.

**Figure 8:**
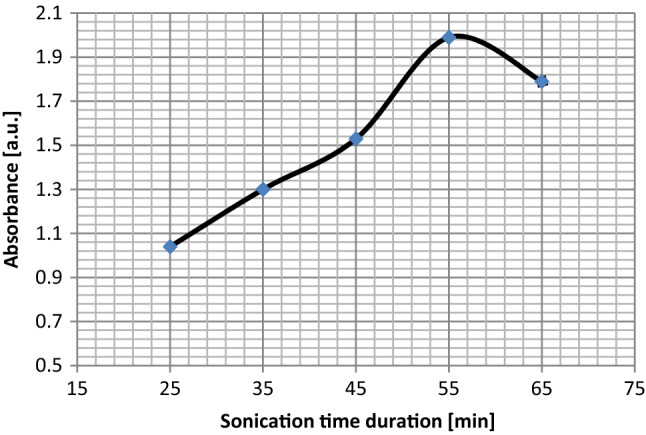
Variation of the UV–visible absorbance of the graphene samples versus sonication time duration.

Influence of the effective time duration of the sonication for graphene dispersions in the water–ethanol solutions at 264 W power is studied to find out the range of graphene flakes production in different sonication time duration of 25, 35, 45, 55, and 65 min, which the results are shown in Fig. 7. From Fig. 8, one can see that the average amount of absorbance increases gradually with an increase in effective sonication times up to 55 min and then decreases. An explanation for the observed result is that prolonged exposure time provides more occasions for delamination of more flakes. However, Above 55 min of exposure, there might be a competition between increasing factors and the tendency for nano-sheets to aggregate with each other. Therefore, one could see that after 55 min, the amount of absorbance is reduced.

**Figure 9:**
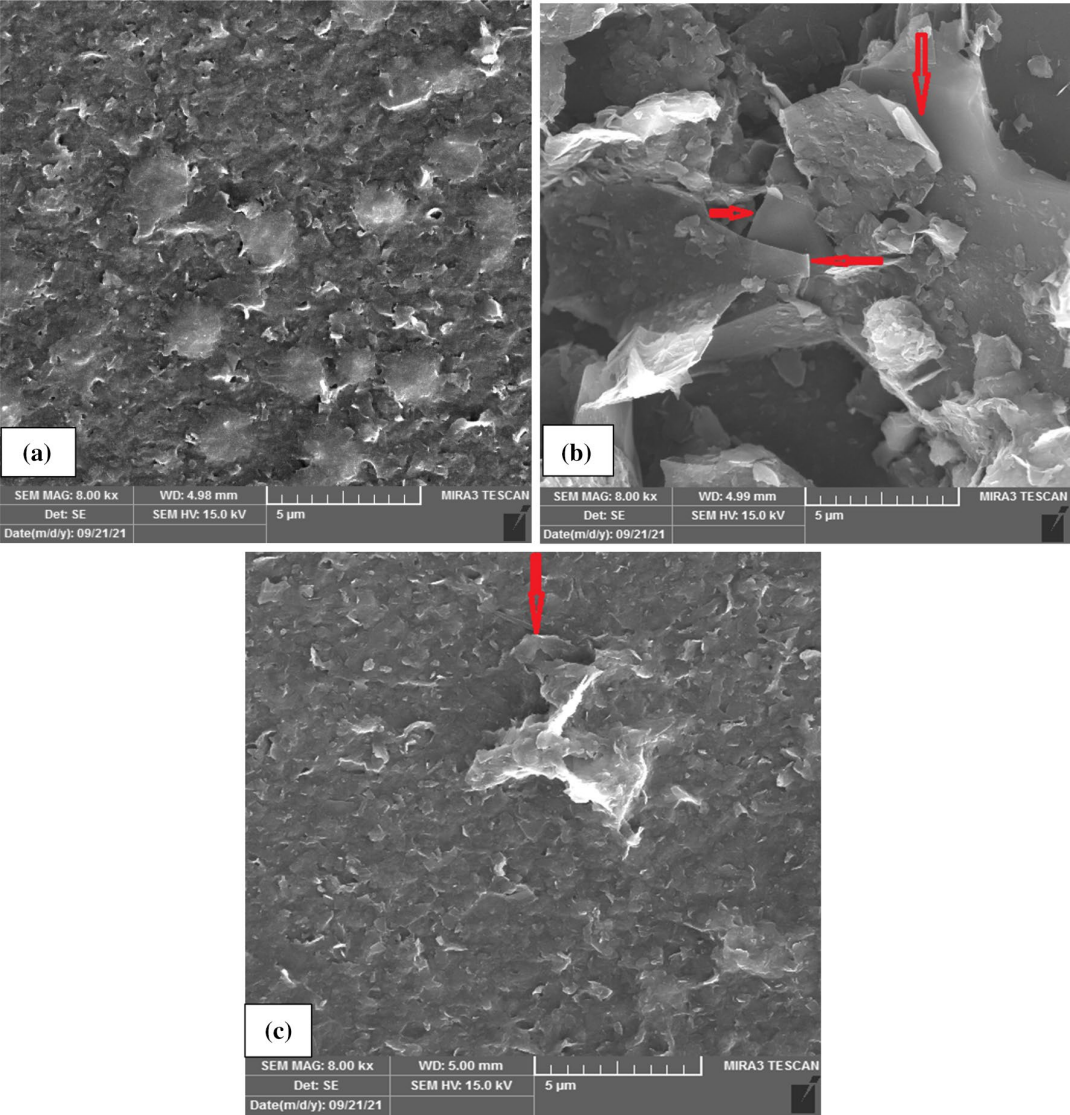
SEM micrographs of graphene flakes for different sonication time duration: (**a**) 25 min, (**b**) 55 min and (**c**) 65 min (scale bars is 5 µm).

The SEM micrographs shown in Fig. 9 confirm the results mentioned above. As can be seen, the flakes in Fig. 9b for 55 min are more prominent and thinner than the flakes in Fig. 9a for 25 min of sonication. By increasing the effective sonication time from 25 to 55 min, more exfoliation occurs. But, above 55 min, the quality of the flakes decreases because of the agglomeration process. Simultaneous agglomeration and crush are observed in Fig. 9c in the flakes.

All findings in this research result in the fact that our most delicate graphene samples in these experimentations are acquired at; power of 264 W, pulse duration of 50% and 55 min irradiation duration which is the “optimum situation” for producing graphene flakes.

**Figure 10:**
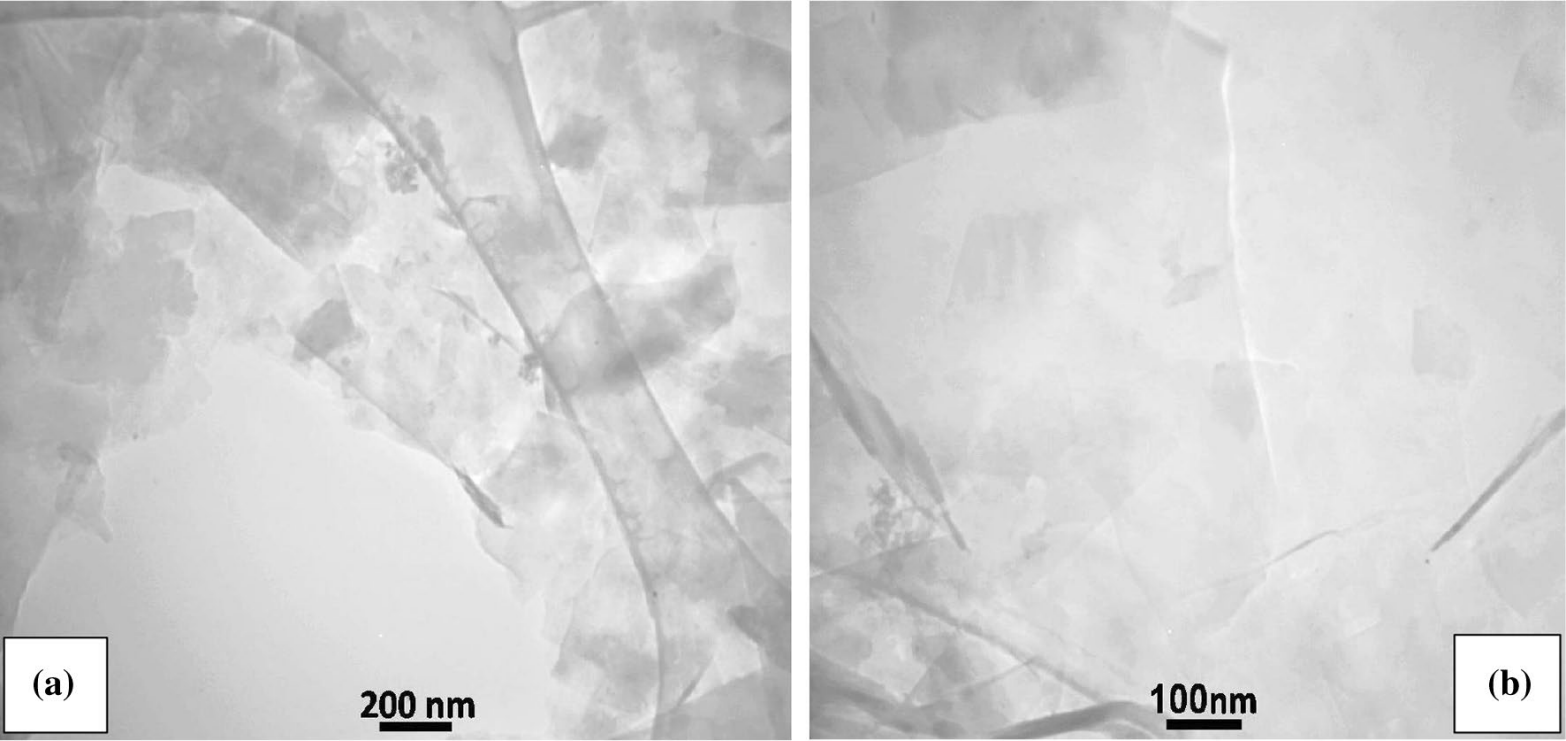
TEM micrographs of the exfoliated graphene flakes, scale bar: (**a**) 200 nm, (**b**) 100 nm.

The structural characterization of the graphene nano-sheets is further examined by TEM (Zeiss EM10C, 100kv). Figure 10a shows that this suspension contained well-exfoliated graphene sheets, including multilayers (less than10 layers) and some few layers (1–3 layers), with larger sheets (Fig. 10b). Figure 10a shows clearly partially exfoliated graphite flakes with some aggregation and also multilayer graphene sheets which looks darker gray. The few-layer graphene sheets with thin flat graphene flakes in large dimensions are shown in Fig. 10b (also Fig. 9b). The results indicate that the exfoliated graphene sheets are few-layer without significant structural defects so this process could be scaled up successfully.

**Figure 11:**
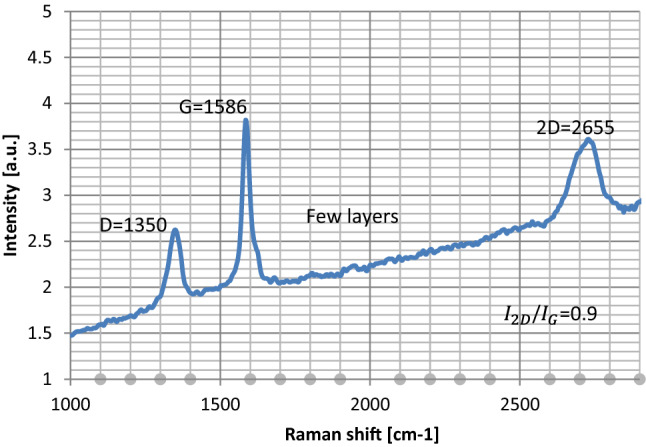
Raman spectrum of dispersed few layer graphene flake exfoliated using ultrasonic sonication.

For more reassurance, the analysis of Raman spectroscopy is used. Figure 11 shows the Raman spectrum of synthesized FLG. In the Raman spectrum of graphene, three distinct features are prominently named the D peak at ~ 1350 cm^−1^, the G peak at ~ 1586 cm^−1^, and the 2D peak at ~ 2655 cm^−1^ are existing. Therefore, Raman spectrum of the sample as shown in Fig. 11 conform the formation of the few layer graphene. ${I}_{2D/{I}_{G}}$ ratio of the sample can be calculated from the observed peaks. Based on the ${I}_{2D/{I}_{G}}$ ratio from the sample’s Raman spectra is calculated to be about 0.95–1. According to the previous report [10], the number of the layers can be derived from the ratio of the peak intensities, ${I}_{2D/{I}_{G}}$, as well as the position and the shape of these peaks. The ${I}_{2D/{I}_{G}}$ ratio being ~ 2 to 3 is for monolayer, ratio being ${2>I}_{2D/{I}_{G}}>1$ for bilayer graphene and ratio being ${I}_{2D/{I}_{G}}<1$ for multilayer graphene. Therefore, it can be concluded that the graphene which are produced in the present report are few layers due to ${I}_{2D/{I}_{G}}$ ratio being ~ 1.

## Conclusion

We studied the effects of the critical ultrasonic LPE parameters, i.e., power and time duration of sonication, on the quality and yield of FLG flakes produced in DI Water–Ethanol in a vessel with 150 mL of water/ethanol-powder solution. In conclusion, graphene produced by sonication is pure graphene and does not need reduction from GO or purification by washing out chemicals. A distribution of acoustic pressure events throughout the treated volume assisted by acoustic pressure difference is a critical factor in producing more quantity and high-quality FLG flakes. The numerical simulations approve the experimental results, which is an increase in the sonicator radiation power increases the conversion of graphite to graphene due to an increase in pressure differences. In addition, for 264 W of sonicator power, the shift in absorption spectra from 266 to 270 nm occurs, which reveals graphene exfoliation with few-layer (1–3 layers). The UV–visible technique is proven to be very effective in studying the number of layers exfoliated and detecting the difference in conjugated polyenes that affect π to π* plasmon peak.

Characterization by UV–visible, SEM, TEM, and Raman spectrum shows that the few-layer graphene for sonication time of 55 min is the optimum for high-quality pure graphene exfoliated from graphite powder for this setup. Graphene with a sonication time of 55 min has a thinner and larger sheet compared to sonication for less or more time duration. Therefore, our findings from the analyses presents the optimum operating parameters for sonicator with the probe of 22 mm in diameter and pulse mode being 50% with 250 mg of graphite in the 125 ml of water–ethanol solution and 150 mL beaker to achieve few-layer graphene flakes with good yield are obtained at 264 W of power, and 55 min of sonication time duration.
